# Investigation of Risk Factors for Postoperative Delirium after Transcatheter Aortic Valve Implantation: A Retrospective Study

**DOI:** 10.3390/jcm11123317

**Published:** 2022-06-09

**Authors:** Yuko Ogata, Naoya Kobayashi, Masanori Yamauchi

**Affiliations:** Department of Anesthesiology and Perioperative Medicine, Tohoku University Graduate School of Medicine, 2-1 Seiryomachi, Aoba, Sendai 980-8575, Miyagi, Japan; yukokosegawa@gmail.com (Y.O.); yamauchi@med.tohoku.ac.jp (M.Y.)

**Keywords:** transcatheter aortic valve implantation, aortic stenosis, postoperative delirium

## Abstract

Transcatheter aortic valve implantation (TAVI) is an effective treatment for severe aortic stenosis (AS); however, postoperative delirium (POD) can worsen patient outcomes. This study aimed to examine the risk factors for POD after TAVI, including possible intervening factors. We included 87 patients (mean age: 83) who underwent TAVI between May 2014 and September 2018. POD was defined by the presence or absence of delirium on ICU admission, assessed using the Confusion Assessment Method for the ICU. Factors that showed significant differences in the univariate analysis were analyzed using a multiple logistic regression analysis. In total, 31 patients (36%) had POD after ICU admission, and 56 (64%) did not. The preoperative frailty score and aortic valve opening area (AVA) were significant risk factors for POD. The multivariate analysis also showed that both factors were independent risk factors for POD (area under the receiver operating characteristic curve: 0.805). There were no significant differences in the number of ICU days. However, postoperative hospitalization was significantly longer in the POD group (19 (17–31) days vs. 16 (13–22) days; *p* = 0.002). POD was associated with a narrow AVA and frailty; this suggests that frailty prevention interventions according to the AVA may be important.

## 1. Introduction

In recent years, transcatheter aortic valve implantation (TAVI) has been widely used to treat severe aortic stenosis (AS) in the older people. Its results are comparable to or better than those of open-heart surgery. The indications for the procedure, which had been limited to older, high-risk patients, have expanded with the evolution of devices and the stability of the procedure, and TAVI has proven to be non-inferior to surgical treatment in low- and moderate-risk patients [1].

However, postoperative delirium (POD), which has shown a considerably lower incidence after TAVI (10–44%) than after open-heart surgery (>50%) [2,3,4,5,6], remains a cause of poor outcomes, including longer intensive care unit (ICU) stays [5], hospital stays [7], and increased mortality [5,8]. Many risk factors for POD have been reported because of patient background factors, such as advanced age, male sex [5], preoperative cognitive function [9], sedatives and other medications [10], and multiple organ damage [9,11]. However, few factors are available for intervention. The dilemma is that patients undergoing general surgery may benefit from increased preoperative activity. However, patients with AS are limited or prohibited from exercise therapy depending on disease severity. It is necessary to identify which preoperative tests as indicators of AS severity influence POD and then integrate these tests to determine which functions can be improved to prevent POD.

Therefore, this study aimed to investigate the risk factors for POD after TAVI. The present findings could help to explore intervention methods for the prevention of POD according to severity.

## 2. Materials and Methods

### 2.1. Study Design and Population

This retrospective observational study was conducted at Tohoku University Hospital. Ethical approval was obtained from Tohoku University Graduate School of Medicine Ethics Committee (reference number: 2018-1-183). The requirement for informed consent was waived owing to the study‘s retrospective nature. The study included all patients who underwent TAVI under general anesthesia between May 2014 and September 2018. Indications for TAVI were any of the following: severe AS with positive clinical symptoms, age ≥80 years, frailty, Society of Thoracic Surgeons score >4%, difficulty in open-heart surgery, previous open-heart surgery, previous chest radiation therapy, deformity or scoliosis of the rib cage, and difficulty in blocking the ascending aorta. Patients with the following criteria were excluded from the study: infective endocarditis, severe stenosis of coronary arteries, presence of other diseases requiring open-heart surgery, difficult vascular access, weakness, and poor morphology of valve rings.

### 2.2. Preoperative Geriatric Assessment

The Mini-Mental State Examination (MMSE) was used to assess preoperative cognitive functions. The frailty index based on the Cardiovascular Health Study (CHS) Index was used to assess frailty [12]. The questionnaire was a self-administered questionnaire consisting of five items, namely, nutrition, fatigue, walking speed, physical activity, and cognitive functions (Supplementary Materials Table S1). Three or more items were considered frail, and one or two items were considered prefrail. If the patient could not write, a Physical Therapist (PT) wrote on his behalf.

### 2.3. Preoperative Cardiac Function Assessment

Preoperative cardiac functions were evaluated using the New York Heart Association (NYHA) classification and echocardiographic findings. Laboratory technicians preoperatively performed transthoracic echocardiography for all patients who presented to our hospital with severe AS either in the ultrasound laboratory or at the bedside. Left ventricular systolic function was evaluated using left ventricular systolic ejection fraction (LVEF) based on left ventricular end-diastolic volume and left ventricular end-systolic volume using the modified Simpson method. Left ventricular diastolic capacity was evaluated using the flow velocity ratio (E/A) based on the maximum velocity of the early diastolic wave (E wave) and atrial systolic wave (A wave) of the left ventricular inflow velocity waveform. The decay time of the E wave (DcT) was used. The maximum velocity of the mitral annulus in early diastole (e’) was measured using the tissue Doppler method to evaluate the left ventricular relaxation capacity, and E/e’ was calculated to estimate the mean left atrial pressure.

To evaluate AS, we used the maximum and mean left ventricular–aortic pressure gradient calculated from the valve currency blood flow velocity by continuous-wave Doppler and the valve opening area calculated from the continuous equation. A low-dose dobutamine-loading echocardiography was performed at the cardiologist’s discretion for patients with reduced LVEF and low-flow low-pressure gradient AS to determine the indication for surgery. Surgical or percutaneous valve replacement indications in severe and very severe cases were discussed based on the echocardiography results.

### 2.4. Assessment and Treatment of POD

Upon admission to the ICU, the nurse assessed the patient’s state of consciousness using the Richmond Agitation–Sedation Scale (RASS), and patients with an RASS score from −3 to +4 continued to be assessed for POD using the Confusion Assessment Method for the ICU (CAM-ICU). Patients who could not be assessed using the CAM-ICU were assessed as soon as they reached an RASS score ≥−3. After ICU admission, the patients were divided into two groups according to the initial CAM-ICU: POD and non-POD groups.

### 2.5. Data Collection

A geriatric assessment was conducted by a PT at the bedside. All study variables were retrieved from an institutional computerized electronic medical recording system (PrimeGaia^®^ (Nihon Kohden Corporation, Tokyo, Japan) and HOPE/EGMAIN-GX^®^ (FUJITSU Corporation, Tokyo, Japan)). No identifying information was recorded to ensure patient confidentiality.

### 2.6. Outcomes

The primary outcome was a positive CAM-ICU score after ICU admission. The secondary endpoints were preoperative cognitive functions, echocardiographic parameters, operative factors, ICU stay, and hospital stay.

### 2.7. Statistical Analysis

Data analyses were performed using JMP 15 software (SAS Institute Inc., Cary, NC, USA). The Wilcoxon test and Fisher’s exact test were used, as appropriate, to compare the data between the two groups. Statistical significance was set at *p* < 0.05. Data are reported as the mean ± SD, unless otherwise indicated. Preoperative factors that showed significant differences in the univariate analysis were analyzed using a multiple logistic regression analysis. In this study, we aimed to predict POD occurrence only with explanatory factors up to the time before the patient entered the operating room, in order to find factors that could allow preoperative interventions to be performed. Even when explanatory factors did not reach statistical significance in the multivariate analyses, they were excluded from the analyses and analyzed again in order to reduce the number of explanatory factors. A post hoc analysis was conducted to determine whether the existing dataset contained the appropriate number of cases to determine significant differences. Multicollinearity was assessed using the variance inflation factor. A variance inflation factor exceeding 10 indicates serious multicollinearity, and values greater than 4.0 may cause concern. The conformity to a linear gradient was graphically verified, and polynomial or logarithmic transformations were performed when necessary. The area under the receiver operating characteristic curve (AUROC) was used to compare the accuracy, which was evaluated as low (0.5–0.7), moderate (0.7–0.8), and high (≥0.8).

## 3. Results

All the patients who underwent TAVI during the study period were included in the analysis. None of the patients met the predefined exclusion criteria. Ultimately, data from the 87 patients who met the inclusion criteria were analyzed. In the post hoc analysis, the number of cases was sufficient (alpha = 0.05, effect size = 0.15, critical t = 1.663, power = 0.974). In total, 31 patients (36%) had POD after ICU admission, and 56 (64%) did not. Patient characteristics are shown in Table 1. There were no differences in profile data such as patient age, sex, and body mass index between the two groups. In terms of severity, the POD group had significantly worse frailty scores (POD group median, 4 (quartile range; 3, 5) vs. non-POD group, 2 (1,3); *p* < 0.001), MMSE (25 (22, 27) vs. 27 (24, 29); *p* = 0.020), European System for Cardiac Operative Risk Evaluation (EURO) score (6.1 [4.5, 10.0] vs. 3.8 [2.9, 5.5]; *p* = 0.006), and NYHA score. The NYHA score (*p* = 0.002) was significantly worse in the POD group (Table 1).

In terms of patient outcomes, the POD group had a longer postoperative hospital stay (19 days (17, 31) vs. 16 days (13, 22]; *p* = 0.002). There were no statistically significant differences in ICU and HCU length of stay, and there were no deaths. On preoperative echocardiography, the aortic valve opening area (AVA) (0.56 [0.45–0.62] vs. 0.69 [0.58–0.84]; *p* < 0.001) and aortic valve opening area index (AVAI) (0.42 [0.35–0.50] vs. 0.51 [0.42–0.60]; *p* = 0.020) were worse in the POD group (Table 2). With regard to surgery, the POD group had a higher rate of inhalation anesthetic use (54.8% vs. 23.2%; *p* = 0.003) and longer surgery time (153 (130, 213) vs. 141 (122, 160) minutes; *p* = 0.002; Table 3).

**Figure 1:**
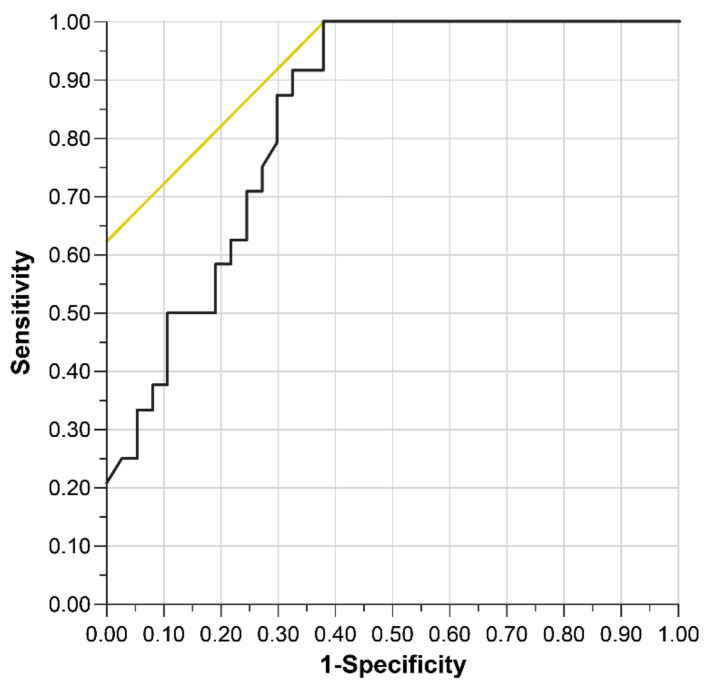
Receiver operating characteristic curve for prediction of POD.

We performed a multivariate analysis of preoperative risk factors for POD. In the univariate analysis, statistically significant differences were found in the frailty index, MMSE, EURO score, NYHA, and GFR among patient backgrounds. Among echocardiographic measures, these were the AVA and AVAI. Considering the frailty index and MMSE, which were the same objective endpoints, the MMSE score was excluded from the multivariate analysis because the difference was less significant. Similarly, between the AVA and AVAI, the AVAI was excluded. In the multivariate analysis, no statistically significant differences were found except for the frailty index and AVA, which were excluded from the final model. The results show that the AVA (odds ratio: 1.95 (per −0.1 cm^2^); 95% confidence interval (CI): 1.17–3.27; *p* = 0.004) and frailty index (odds ratio: 2.49 (per +1 point); 95% CI: 1.37–4.54; *p* < 0.001) were independent risk factors (Table 4). The AUROC of the multivariate model was 0.840, the R-squared value (R^2^) was 0.321, and Akaike’s Information Criterion (AIC) was 61.9 (Figure 1).

The receiver operating characteristic (ROC) curve represents the sensitivity and specificity minus one on the x-axis and y-axis, respectively. The test accuracy depended on how well the test group could be divided into those with and without POD. Accuracy is measured by the area under the curve of the ROC (AUROC): an area of 1 represents a perfect test, and an area of 0.5 represents an inconclusive test. R^2^ = 0.321, AUROC = 0.840, and Akaike’s information criterion = 61.9. POD: postoperative delirium.

## 4. Discussion

In this study, a narrow AVA and frailty were shown to be independent risk factors for POD. It is important to note that these two factors predicted POD with a probability as high as 0.840 for the AUROC.

### 4.1. The Narrow AVA

POD is a frequently observed complication after cardiovascular surgery that develops acutely, is associated with a decline in consciousness, attention, and cognition, and is closely related to prolonged ICU stays, longer hospital stays, and increased mortality. Both patient-specific factors (e.g., older age, male sex, cognitive dysfunction, and frailty) and intervening factors (surgery and associated complications) have been reported as contributing factors [5,13,14,15,16,17].

It is empirically known that POD increases with the severity of illness in patients with AS; however, there are insufficient discussions on which value should be used from multiple severity indices. Previous reports have reported an increase in the odds ratio of 2.39 when the AVA fell below 0.75 cm^2^ [5], but no clear rationale exists for using this value as a cutoff. In this study, the continuous interpretation of the AVA showed that the odds ratio increased by 1.95 for every 0.1 cm^2^ decrease. It also showed that the narrower the AVA was, the greater POD was. Furthermore, other than the AVA, preoperative laboratory data, including the LVEF, showed little relationship with POD, suggesting that the AVA is a more important indicator.

The mechanism by which a narrow AVA induces POD is thought to be reduced cardiac output, which in turn causes cardiac remodeling and ultimately reduces left ventricular contractility and cerebral blood flow [18,19]. Furthermore, a narrow AVA reduces the accessibility of TAVI, which increases the complexity of the procedure, including the need for pre-dilation and post-dilation and repositioning. It may lead to the possibility of cerebral micro-emboli as well as impaired neuro-prognosis and cognitive functions [20].

### 4.2. Frailty

In this study, preoperative frailty was an independent risk factor for POD. Frailty is an important factor that can be addressed in the preoperative period. A meta-analysis reported in 2021 showed a significant increase in POD in wait-listed surgical patients aged >65 years with frailty, especially in patients with AS [21]. Although preoperative frailty has been shown to be related to POD even in patients undergoing TAVI [22,23], there are more than 20 reported methods for assessing frailty, and the lack of uniform criteria is a challenge. The frailty index, a self-administered questionnaire based on the criteria of the CHS used in this study, is one of the most common methods and can easily assess frailty with only five questions (Supplementary Materials Table S1) [12]. It is noteworthy that, despite the simplicity of the assessment method, POD risk could be easily discriminated against, even after adjusting for the AVA in this study.

Frailty is considered reversible [24,25], and both exercise and non-exercise therapies are used for its prevention [26]. Non-exercise therapy includes nutritional support, hormone therapy, and cognitive training. Recent guidelines for managing frailty recommend exercise therapy and adequate protein intake as non-exercise therapy [27]. In contrast, severe AS, which is an indication for surgery, is an absolute contraindication to active exercise therapy for symptomatic patients and a relative contraindication for the asymptomatic ones [28]. However, non-exercise therapy can be aggressively implemented, especially nutritional therapy recommended by the guidelines, which should be sufficiently intervened upon before TAVI is performed. In conclusion, for the effective prevention of frailty, the early assessment of AS frailty may be useful, before it becomes an indication for surgery or before symptoms appear, as may be the implementation of exercise and nutritional therapy according to the AVA. For patients with AS exhibiting a narrowed AVA, the risk of POD should be considered. Additionally, emphasis should be placed on nutrition, the maintenance of anesthesia depth, the selection of non-narcotic analgesics, early POD monitoring, pain control, and early postoperative non-athletic interventions, such as exercise rehabilitation and cognitive training [29,30].

### 4.3. Limitations

This was a single-center observational study. POD was determined in the CAM-ICU, and low-activity delirium may not have been detected. In addition, the proportion of female patients with longevity was higher in both groups due to the higher age of indication for national TAVI. POD is considered more common in male patients, which may be the reason for the lack of significant differences. The frailty index, a criterion for evaluating frailty, cannot disregard the possibility that the subjectivity of the patients may have influenced the results. Future studies involving interventions may use tools that include more objective assessment items. Future studies should include multicenter, larger, prospective studies; the introduction and evaluation of several different frailty assessment tools; and the implementation of several preoperative interventions to confirm their effectiveness, especially for the AVA and frailty.

## 5. Conclusions

This study suggests that POD is associated with a narrow AVA and frailty and that frailty prevention interventions according to the AVA may be important.

## Figures and Tables

**Table 1:** Patient characteristics.

	POD ^1^		Non-POD		*p*-Value	
*N*	31		56			
Age, median (years)	84	(80, 88)	83	(81, 86)	0.759	
Female	25	(80.6 %)	40	(71.4 %)	0.344	
Body mass index (kg/m²)	22.7	(20.3, 24.7)	22	(20.5, 23.6)	0.47	
Severity scores						
Frailty index	4	(3, 5)	2	(1, 3)	<0.001	**
MMSE ^2^	25	(22, 27)	27	(24, 29)	0.02	*
STS ^3^ score	6.7	(5.3, 10.9)	6	(4.5, 8.4)	0.125	
EURO ^4^ score	6.1	(4.5, 10.0)	3.8	(2.9, 5.5)	0.006	**
NYHA ^5^					0.002	**
I	0	(0 %)	0	(0 %)		
II	13	(14.9 %)	41	(73 %)		
III	14	(16.1 %)	15	(27 %)		
IV	4	(4.6 %)	0	(0 %)		
Comorbidities						
Hypertension	22	(25.3 %)	43	(76.8 %)	0.55	
Atrial fibrillation	2	(2.3 %)	2	(3.6 %)	0.539	
Pacemaker implantation	1	(1.1 %)	3	(5.4 %)	0.649	
Diabetes mellitus	9	(10.3 %)	12	(21.4 %)	0.427	
Stroke	1	(1.1 %)	5	(8.9 %)	0.315	
Myocardial infarction	2	(2.3 %)	2	(3.6 %)	0.539	
PCI ^9^	7	(8 %)	10	(17.9 %)	0.595	
Angina	0	(0 %)	4	(7.1 %)	0.128	
Dementia	2	(2.3 %)	0	(0 %)	0.055	
COPD ^10^	3	(3.4 %)	3	(5.4 %)	0.446	
Carotid artery disease	1	(1.1 %)	3	(5.4 %)	0.649	
Rheumatoid arthritis	2	(2.3 %)	2	(3.6 %)	0.539	
Current smoker	6	(6.9 %)	16	(28.6 %)	0.344	
Habitual drinking	2	(2.3 %)	11	(20 %)	0.098	
Use of sleeping pills	9	(10.3 %)	18	(32.1 %)	0.764	
Physiological variables						
Albumin (g/L)	3.6	(3.2, 3.8)	3.7	(3.4, 4)	0.07	
pre-BNP ^7^ (pg/dL)	203.2	(93.4, 603.2)	178	(78, 425.8)	0.257	
GFR ^8^ (mL/mL/1.73mm²)	42	(35, 56)	53	(40, 77)	0.021	*
FEV ^6^ 1.0 < 70%	9	(29 %)	8	(14.3 %)	0.097	
Outcomes						
ICU-free days 30days (days)	28	(27, 28)	28	(27.3, 28.6)	0.258	
Postoperative hospital stays (days)	19	(17, 31)	16	(13, 22)	0.002	**
Mortality	0	(0 %)	0	(0 %)	1	

Patient characteristics in the POD and non-POD groups are shown separately. Values represent the number of patients (rates) or median (interquartile range). The Wilcoxon test and Fisher’s exact test were used to compare data between POD and non-POD patients. * *p* < 0.05, ** *p* < 0.01. ^1^ POD, postoperative delirium; ^2^ MMSE, Mini-Mental State Examination; ^3^ STS, Society of Thoracic Surgeons; ^4^ EURO, European System for Cardiac operative Risk Evaluation; ^5^ NYHA, New York Heart Association; ^6^ FEV, forced expiratory volume in one second; ^7^ BNP, brain natriuretic peptide; ^8^ GFR, glomerular filtration rate; ^9^ PCI, peri-cutaneous coronary intervention; ^10^ COPD, chronic obstructive pulmonary disease.

**Table 2:** Preoperative transthoracic echocardiography.

	POD ^4^		Non-POD		*p*-Value	
*N*	31		56			
Aortic valve						
Peak jet velocity (m/s)	5.0	(4.6, 5.3)	4.6	(4.2, 5.4)	0.115	
Maximal gradient (mmHg)	100	(83, 113)	83	(71, 119)	0.133	
Mean gradient (mmHg)	56	(46, 67)	50	(39, 70)	0.226	
AVA ^1^ (cm^2^)	0.56	(0.45, 0.62)	0.69	(0.58, 0.84)	<0.001	**
AVAI ^2^ (cm^2^/m^2^)	0.42	(0.35, 0.50)	0.51	(0.42, 0.60)	0.020	*
Left ventricular function						
Ejection fraction (%)	64	(57, 71)	65	(57, 73)	0.529	
End-diastolic volume (mL/m^2^)	47	(44, 51)	46	(41, 50)	0.401	
End-systolic volume (mL/m^2^)	30	(27, 33)	29	(25, 34)	0.356	
Left ventricular expandability						
DcT ^3^ (msec)	201	(168, 303)	235	(181, 310)	0.643	
E/A	0.7	(0.5, 0.9)	0.6	(0.5, 0.8)	0.598	
E/e’	19.3	(15.8, 27.4)	19.7	(15.6, 24.0)	0.793	

The risk factors for POD are shown separately. Values are expressed as medians (interquartile ranges). The Wilcoxon test was used to compare the data between POD and non-POD patients. * *p* < 0.05, ** *p* < 0.01. ^1^ AVA, continuity equation valve area; ^2^ AVAI, continuity equation valve area index; ^3^ DcT, deceleration time of early diastolic filling velocity wave; ^4^ POD, postoperative delirium.

**Table 3:** Intraoperative factors of POD.

	POD ^1^		Non-POD		*p*-Value	
*N*	31		56			
Anesthesia						
Sedative agents					0.003	**
Gas	17	(54.8 %)	13	(23.2 %)		
Total intravenous anesthesia	14	(45.2 %)	43	(76.8 %)		
Anesthesia time, median (IQR) ^2^	286	(260, 340)	262	(244, 283)	0.002	**
Operation time, median (IQR)	153	(130, 213)	141	(122, 160)	0.019	*
Time from start of surgery to dilation (IQR)	81	(65, 107)	75	(61, 94)	0.087	
Operation						
Approach					0.756	
Trans-femoral	28	(90.3 %)	51	(91.1 %)		
Trans-apical	1	(3.2 %)	3	(5.4 %)		
Subclavian	2	(6.5 %)	2	(3.6 %)		
Device					0.293	
CoreValve^TM^/Evolut^TM^ R	15	(48.4 %)	23	(41.1 %)		
Edwards SAPIEN	16	(51.6 %)	33	(58.9 %)		
Prosthesis size	26	(83.9 %)	26	(46.4 %)	0.348	

The risk factors for POD are shown separately. Values represent the number of patients (rates) or median (interquartile range). The Wilcoxon test and Fisher’s exact test were used to compare data between POD and non-POD patients. * *p* < 0.05, ** *p* < 0.01. ^1^ POD: postoperative delirium, ^2^ IQR: Inter-quartile range.

**Table 4:** Risk factors of postoperative delirium.

	Odds Ratio	95% CI	*p*-Value	
Aortic valve area (per −0.1) (cm^2^)	1.95	(1.17–3.27)	0.004	**
Frailty index (per +1)	2.49	(1.37–4.54)	<0.001	**

Factors that showed significant differences in the univariate analysis were analyzed using multiple logistic regression analysis. R^2^: 0.321; area under the curve of receiver operating characteristic (AUROC): 0.840; Akaike’s Information Criterion (AIC): 61.9. ** *p* < 0.01. CI: confidence interval.

## Data Availability

Not applicable.

## Supplementary Materials

The following supporting information can be downloaded at: https://www.mdpi.com/article/10.3390/jcm11123317/s1, Table S1: Frailty index based on the Cardiovascular Health Study (CHS) Index.

## References

[1] Van der Wulp K., Van Wely M., Van Heijningen L., Van Bakel B., Schoon Y., Verkroost M., Gehlmann H., Van Garsse L., Vart P., Kievit P. (2019). Delirium After Transcatheter Aortic Valve Implantation Under General Anesthesia: Incidence, Predictors, and Relation to Long-Term Survival. J. Am. Geriatr. Soc..

[2] Tse L., Bowering J.B., Schwarz S.K., Moore R.L., Burns K.D., Barr A.M. (2015). Postoperative delirium following transcatheter aortic valve implantation: A historical cohort study. Can. J. Anaesth..

[3] Bagienski M., Kleczynski P., Dziewierz A., Rzeszutko L., Sorysz D., Trebacz J., Sobczynski R., Tomala M., Stapor M., Dudek D. (2017). Incidence of Postoperative Delirium and Its Impact on Outcomes After Transcatheter Aortic Valve Implantation. Am. J. Cardiol..

[4] Eide L.S., Ranhoff A.H., Fridlund B., Haaverstad R., Hufthammer K.O., Kuiper K.K., Nordrehaug J.E., Norekvål T.M. (2015). Comparison of frequency, risk factors, and time course of postoperative delirium in octogenarians after transcatheter aortic valve implantation versus surgical aortic valve replacement. Am. J. Cardiol..

[5] Mauri V., Reuter K., Körber M.I., Wienemann H., Lee S., Eghbalzadeh K., Kuhn E., Baldus S., Kelm M., Nickenig G. (2021). Incidence, Risk Factors and Impact on Long-Term Outcome of Postoperative Delirium After Transcatheter Aortic Valve Replacement. Front. Cardiovasc. Med..

[6] Hoogma D.F., Venmans E., Al Tmimi L., Tournoy J., Verbrugghe P., Jacobs S., Fieuws S., Milisen K., Adriaenssens T., Dubois C. (2021). Postoperative delirium and quality of life after transcatheter and surgical aortic valve replacement: A prospective observational study. J. Thorac. Cardiovasc. Surg..

[7] Milbrandt E.B., Deppen S., Harrison P.L., Shintani A.K., Speroff T., Stiles R.A., Truman B., Bernard G.R., Dittus R.S., Ely E.W. (2004). Costs associated with delirium in mechanically ventilated patients. Crit. Care Med..

[8] Witlox J., Eurelings L.S., de Jonghe J.F., Kalisvaart K.J., Eikelenboom P., van Gool W.A. (2010). Delirium in elderly patients and the risk of postdischarge mortality, institutionalization, and dementia: A meta-analysis. JAMA.

[9] Van Rompaey B., Schuurmans M.J., Shortridge-Baggett L.M., Truijen S., Bossaert L. (2008). Risk factors for intensive care delirium: A systematic review. Intensive Crit. Care Nurs..

[10] Vasilevskis E.E., Han J.H., Hughes C.G., Ely E.W. (2012). Epidemiology and risk factors for delirium across hospital settings. Best Pract. Res. Clin. Anaesthesiol..

[11] Aldemir M., Ozen S., Kara I.H., Sir A., Baç B. (2001). Predisposing factors for delirium in the surgical intensive care unit. Crit. Care.

[12] Yamada M., Arai H. (2015). Predictive Value of Frailty Scores for Healthy Life Expectancy in Community-Dwelling Older Japanese Adults. J. Am. Med. Dir. Assoc..

[13] Singer J., Trollor J.N., Baune B.T., Sachdev P.S., Smith E. (2014). Arterial stiffness, the brain and cognition: A systematic review. Ageing Res. Rev..

[14] Avila-Funes J.A., Meillon C., González-Colaço Harmand M., Tzourio C., Dartigues J.F., Amieva H. (2014). Association between frailty and carotid central structure changes: The Three-City Study. J. Am. Geriatr. Soc..

[15] Lin C.H., Chou C.Y., Liu C.S., Huang C.Y., Li T.C., Lin C.C. (2015). Association between frailty and subclinical peripheral vascular disease in a community-dwelling geriatric population: Taichung Community Health Study for Elders. Geriatr. Gerontol. Int..

[16] Kobayashi N., Nakagawa A., Kudo D., Ishigaki T., Ishizuka H., Saito K., Ejima Y., Wagatsuma T., Toyama H., Kawaguchi T. (2019). Arterial blood pressure correlates with 90-day mortality in sepsis patients: A retrospective multicenter derivation and validation study using high-frequency continuous data. Blood Press. Monit..

[17] Toyama H., Takei Y., Saito K., Mori S., Ui A., Kobayashi N., Tatebe S., Adachi O., Ejima Y., Yamauchi M. (2018). Ventricular Assist Device Implantation in a Patient With Severe Systemic Right Ventricular Failure and Pulmonary Hypertension Secondary to Congenitally Corrected Transposition of Great Arteries. J. Cardiothorac. Vasc. Anesth..

[18] Matsuda H. (2007). Role of neuroimaging in Alzheimer’s disease, with emphasis on brain perfusion SPECT. J. Nucl. Med..

[19] O’Brien J.T. (2007). Role of imaging techniques in the diagnosis of dementia. Br. J. Radiol..

[20] Vermeer S.E., Longstreth W.T., Koudstaal P.J. (2007). Silent brain infarcts: A systematic review. Lancet Neurol..

[21] Gracie T.J., Caufield-Noll C., Wang N.Y., Sieber F.E. (2021). The Association of Preoperative Frailty and Postoperative Delirium: A Meta-analysis. Anesth. Analg..

[22] Abawi M., Nijhoff F., Agostoni P., Emmelot-Vonk M.H., De Vries R., Doevendans P.A., Stella P.R. (2016). Incidence, Predictive Factors, and Effect of Delirium After Transcatheter Aortic Valve Replacement. JACC Cardiovasc. Interv..

[23] Goudzwaard J.A., De Ronde-Tillmans M., El Faquir N., Acar F., Van Mieghem N.M., Lenzen M.J., De Jaegere P.P.T., Mattace-Raso F.U.S. (2019). The Erasmus Frailty Score is associated with delirium and 1-year mortality after Transcatheter Aortic Valve Implantation in older patients. The TAVI Care & Cure program. Int. J. Cardiol..

[24] Junius-Walker U., Onder G., Soleymani D., Wiese B., Albaina O., Bernabei R., Marzetti E. (2018). The essence of frailty: A systematic review and qualitative synthesis on frailty concepts and definitions. Eur. J. Intern. Med..

[25] Fried L.P., Tangen C.M., Walston J., Newman A.B., Hirsch C., Gottdiener J., Seeman T., Tracy R., Kop W.J., Burke G. (2001). Frailty in older adults: Evidence for a phenotype. J. Gerontol. A Biol. Sci. Med. Sci..

[26] Baztán J.J., De la Puente M., Socorro A. (2017). Frailty, functional decline and mortality in hospitalized older adults. Geriatr. Gerontol. Int..

[27] Turner G., Clegg A. (2014). Best practice guidelines for the management of frailty: A British Geriatrics Society, Age UK and Royal College of General Practitioners report. Age Ageing.

[28] Gati S., Malhotra A., Sharma S. (2019). Exercise recommendations in patients with valvular heart disease. Heart.

[29] Kobayashi N., Shiga T., Ikumi S., Watanabe K., Murakami H., Yamauchi M. (2021). Semi-automated tracking of pain in critical care patients using artificial intelligence: A retrospective observational study. Sci. Rep..

[30] Guenther U., Riedel L., Radtke F.M. (2016). Patients prone for postoperative delirium: Preoperative assessment, perioperative prophylaxis, postoperative treatment. Curr. Opin. Anaesthesiol..

