# Supplementary material for: Investigation of Risk Factors for Postoperative Delirium after Transcatheter Aortic Valve Implantation: A Retrospective Study
Source: J Clin Med. 2022 Jun 9;11(12):3317. doi: 10.3390/jcm11123317 (PMC9225478; doi:10.3390/jcm11123317)
Supplement: Supplementary file 1 [file jcm-11-03317-s001.zip › jcm-1731236-supplementary.pdf]

Supplementary Table S1. The Frailty index based on the Cardiovascular Health Study (CHS) Index.

**Question**	**Score**
1) Have you lost 2 kg or more in the past 6 months?	Yes = 1 pt.
2) Do you think you walk slower than before?	Yes = 1 pt.
3) Do you go for a walk for your health at least once a week?	Yes = 1 pt.
4) Can you recall what happened 5 minutes ago	No = 1 pt.
5) In the past 2 weeks, have you felt tired without reason?	Yes = 1 pt.

The scale was adapted from the study by Yamada et al [12].

Decision: ≥3 points, frail; 1–2 points, prefrail; and 0 points, robust.
